# Protein-ligand binding database tools for selecting, visualizing, and comparing interaction thermodynamics using web applications

**DOI:** 10.1016/j.bpr.2026.100296

**Published:** 2026-10-27

**Authors:** Darius Lingė, Daumantas Matulis

**Affiliations:** Department of Biothermodynamics and Drug Design, Institute of Biotechnology, Life Sciences Center, Vilnius University, Vilnius, Lithuania

## Abstract

Protein-small molecule binding thermodynamics and structural recognition are significant challenges in molecular biophysics and difficult to model computationally. To apply AI-machine learning (ML) approaches for rational drug design of small molecules, a sufficiently large database is required that contains reliable and well-organized machine-readable experimental raw data. Current databases contain data on highly diverse proteins and small molecules, but even small chemical changes can cause large differences in binding affinities, the so-called activity cliff. Therefore, we have assembled a database where the proteins, namely carbonic anhydrases (CAs), are similar and the compounds are primary sulfonamides that are structurally closely related. Despite high similarity of compound chemical structures, the affinities span from undetectable to femtomolar.

The database currently contains data on 12 catalytically active human CA isozymes, approximately 800 compounds, and 10,000 interactions by four techniques: fluorescent thermal shift assay, isothermal titration calorimetry, surface plasmon resonance, and inhibition of enzymatic activity. The thermodynamic data are complemented with 138 X-ray crystal structures of the same ligands bound to 6 CA isozymes. Here, we describe the functionality of tools for web users of the Protein-Ligand Binding Database for data visualization and analysis.

## Why it matters

Protein-ligand binding thermodynamics and kinetics are essential parameters in early-stage drug discovery, providing insights into the affinity, selectivity, and mechanism of molecular recognition. The Protein-Ligand Binding Database (PLBD) contains experimental interaction data for the 12 human carbonic anhydrase isozymes and hundreds of ligands. The database has continued to expand and now includes approximately 10,000 unique records. Efficient presentation, visualization, and analysis of such large data sets are crucial for identifying trends and improving the understanding of ligand-protein binding behavior. Here, we describe a suite of web-based tools developed for PLBD users that facilitate data exploration, visualization, and analysis. These applications are accessible through the PLBD-Tools portal at https://tools.plbd.org.

## Introduction

Measurements of protein-ligand binding thermodynamics play a central role in the early stages of drug discovery and in many other biological and biomedical projects that require quantitative characterization of noncovalent interactions.^1^ Beyond determining binding affinity, thermodynamic parameters provide mechanistic insight into the molecular forces governing recognition processes, including hydrogen bonding, electrostatic interactions, hydrophobic effects, and conformational changes. Such information is valuable for rational lead optimization, improving target selectivity, and understanding the energetic determinants of molecular recognition.

Recent reviews have emphasized that both thermodynamic and kinetic aspects of ligand binding are increasingly recognized as important contributors to the efficacy and developability of drug candidates.^2^ Experimental determination of these parameters, therefore, remains an essential component of contemporary medicinal chemistry and chemical biology workflows. Isothermal titration calorimetry (ITC), thermal shift assay (TSA), and complementary biophysical techniques enable the complete characterization of protein-ligand interactions and have become indispensable tools for structure-activity and structure-thermodynamics relationship studies.

Carbonic anhydrases (CAs) have long served as a model system for investigating protein-ligand recognition, and extensive thermodynamic data sets have demonstrated the utility of intrinsic binding thermodynamics in understanding isoform selectivity and guiding ligand optimization.^3^^,^^4^^,^^5^ Usually, chemical compounds with similar structures bind to CA isozymes with similar characteristics. However, even the smallest possible chemical alterations, such as a methyl group or an oxygen atom, could change the affinity to a significant and unexpected extent.^6^^,^^7^^,^^8^

The rapid growth of biochemical and biophysical data sets has increased the demand for efficient approaches to data presentation, exploration, and interpretation. Recent reviews have emphasized that effective visualization methods are essential for extracting meaningful information from large, heterogeneous experimental data sets, thereby facilitating pattern recognition, hypothesis generation, and scientific communication.^9^^,^^10^

In addition to conventional numerical tables, visual analytics techniques such as heatmaps, interactive plots, and color-encoded representations can substantially improve the accessibility and interpretability of complex biological data. Advances in visualization technologies have also highlighted their importance in structural and molecular sciences, where graphical representations support the analysis of multidimensional experimental observations and enable intuitive comparison of large numbers of measurements. Consequently, the development of specialized visualization tools represents an important step toward improving the usability of affinity databases and enhancing our understanding of molecular recognition processes.

## Materials and methods

### Biophysical methods and procedures

Recombinant proteins were prepared using standard procedures as previously described.^11^^,^^12^ Protein amino acid sequences and various analytical parameters for each purification batch are included in the Protein-Ligand Binding Database (PLBD).

Chemical compounds were synthesized as previously described.^13^^,^^14^

Four techniques were used to determine protein: ligand binding thermodynamics and kinetics, with the values listed in the PLBD; TSA; ITC, surface plasmon resonance (SPR), and enzymatic inhibition assay (SFA). Technique advantages and limitations have been previously described.^15^

X-ray crystal structures were determined as previously described.^15^^,^^16^^,^^17^

### Protein classification in the PLBD

In the PLBD schema accessible at https://plbd.org/doc/db/schemas/main-database-description.xml, protein-related information is distributed across several tables. The “protein” table stores amino acid sequences, while experimental measurements are performed on specific protein batches. Protein purification may introduce minor variations that can affect physical and ligand-binding properties; such batch-specific information is recorded in the “protein_batch” table.

Small sequence modifications, such as His-tags, generally do not significantly influence ligand binding. To account for this, PLBD introduces the concept of a “protein group,” defined as a set of individual proteins and their experimental batches that exhibit equivalent ligand-binding behavior.

In the context of CAs, a protein group corresponds to an isozyme. Accordingly, 12 CA isozyme groups are represented in PLBD: CA I, CA II, CA III, CA IV, CA VA, CA VB, CA VI, CA VII, CA IX, CA XII, CA XIII, and CA XIV. For database table and field naming, these groups are encoded using Arabic numeral-based abbreviations: ca01, ca02, ca03, ca04, ca05a, ca05b, ca06, ca07, ca09, ca12, ca13, and ca14, respectively.

### Informatics methods and procedures

Each web application in the PLBD-Tools collection was designed to serve as a specialized tool for graphical exploration and analysis of the data stored in the existing PLBD tables (Figure 1). The JavaScript Object Notation (JSON) data exchange format for PLBD-Tools was chosen for its popularity and open, structured format.

**Figure 1:**
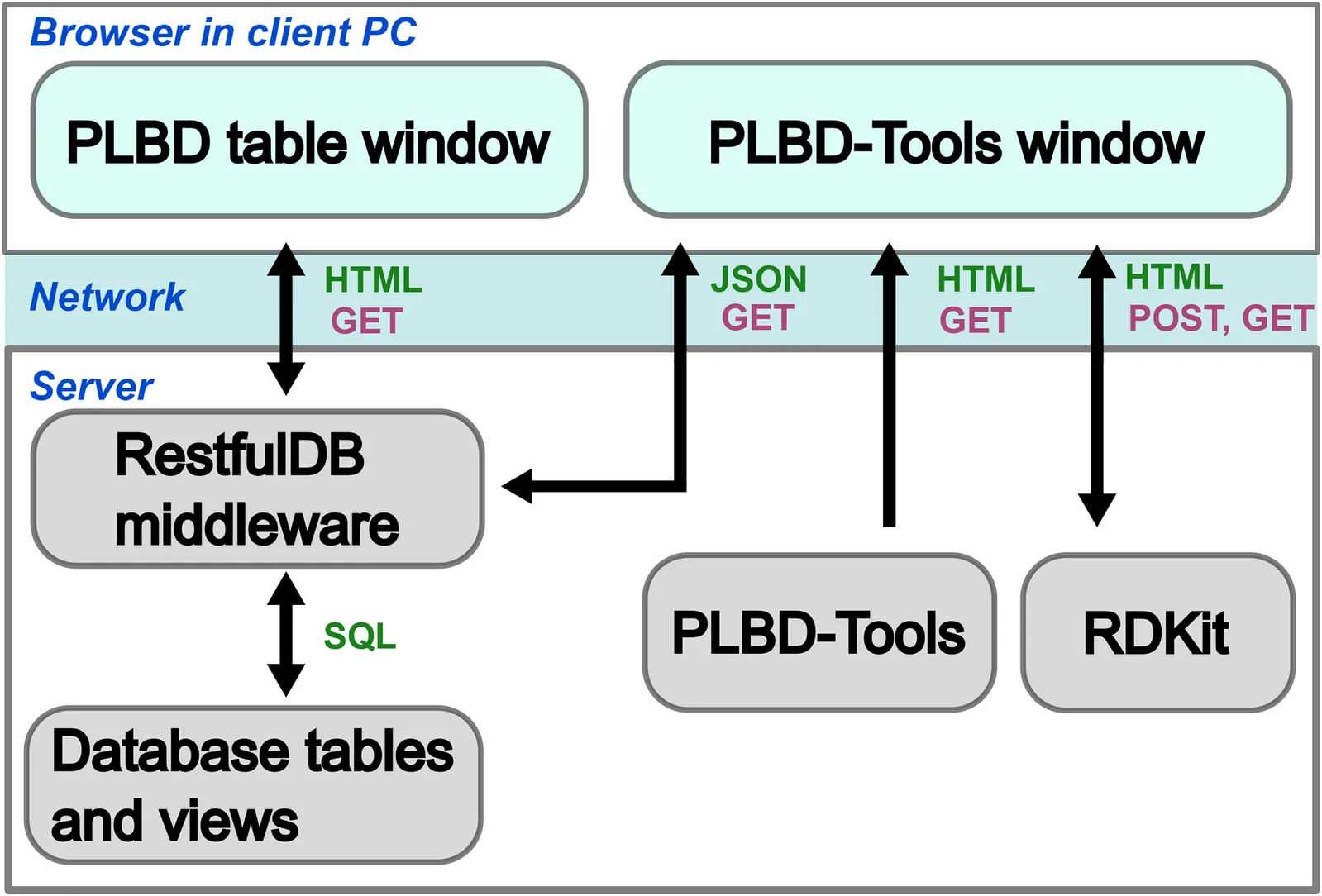
PLBD-Tools position in the Protein-Ligand Binding Database data flow.

#### Web development platform

Contemporary web development platforms often rely on languages such as PHP and Python,^18^^,^^19^ which undergo rapid and continuous version updates. This constant evolution frequently leads to compatibility issues not only across major releases but even between minor versions, resulting in long-term maintenance challenges. On the client side, modern development heavily depends on rapidly evolving frameworks such as React, Angular, and Vue.js, which introduce additional layers of abstraction and increase dependency instability.

To mitigate these risks, this work adopts a simplified client-side approach based on JavaScript as the primary programming language rather than relying on external libraries for the core implementation. JavaScript was selected for its relatively stable execution environment within web browsers. Additionally, the approach avoids the use of transient or rapidly changing libraries such as jQuery in order to minimize external dependencies and improve long-term maintainability. For data visualization, the D3.js library is used as a dedicated external library due to its versatility, expressive power, and strong community support, ensuring both flexibility and sustainability in the proposed solution.

In addition to JavaScript, PLBD-Tools utilizes HTML and CSS. Each web application includes a separate configuration file that defines application-specific variables and their assigned values.

For data exchange with the PLBD server, the JSON format was selected. It enables unambiguous interpretation of textual field records containing complex punctuation (e.g., commas, semicolons, and line-ending symbols). Furthermore, JSON is both human and machine readable and is supported by a wide range of open-source libraries. Another attractive format, CSV, is defined by standards such as RFC 4180 and can represent fields containing commas, line breaks, or carriage returns when properly escaped and quoted. However, in practice, many available CSV libraries and code examples handle such special characters inconsistently or incorrectly, which can lead to ambiguous or erroneous interpretation of field contents. For this reason, CSV was considered less suitable for unpredictable input in the proposed solution.

#### Used external software

• SmilesDrawer.^20^ In the RestfulDB middleware-generated HTML page, loading of each structure image file runs separately. In the table tools, the SmilesDrawer JavaScript component is used to quickly draw structural formulas for compounds from their SMILES strings. This feature reduces data flow between the server and the client device. Besides fast drawing, this approach offers a unified drawing style for structures.
• Chem composer widget from the Kekule.js^21^ JavaScript toolkit is used to generate SMARTS patterns in compound structure fragment search. The widget controls are reduced, concentrating on small structure fragments drawn.
• Bare D3.js^22^ library functions are used for graphics in plot tools.
• SIMPLE STATISTICS^23^ functions are used in plot tools to find the minimal and maximal values of the visible data points and regression line parameters.
• For saving generated SVG files on the web application client side, the FileSaver.js^24^ solution was used.
• To write spreadsheet data to an XLSX file, the ExcelJS^25^ library was used.

#### RDKit package in PLBD-Tools server

For compound structural formulas, drawing, and substructure search, the server was built based on the RDKit^26^ Python implementation and uses the mod_wsgi Apache module with a Python 3 interconnection script. The RDKit package in the PLBD-Tools server is accessible at the address https://tools.plbd.org/rdkit and accepts standard HTTP GET and POST requests.

#### API and PLBD-Tools interconnection

PLBD-Tools are centered around chemical compounds as potential drug candidates, and their interconnection is implemented via a single URL parameter, “compounds.” This parameter defines a set of compound IDs, formatted as a JavaScript array or Python-style set. For example, https://tools.plbd.org/ia.html?compounds=[1783,1857,2355] displays affinity data for acetazolamide, E2, and VD12-23.

When a defined set of compounds is provided via the “compounds” parameter, plot tools display only the corresponding data points by default. These compounds can also be highlighted using the “From URL” list in the “Filters” panel. Disabling the “Only” option reveals all data points from the loaded data set.

## Results

### Results using the PLBD-Tools

We have designed a number of tools to help users analyze data in the PLBD.

• Compound Browser, which enables visualization of the chemical structures of compounds in the database.
• Compound Affinities for CA Isozymes tool, which helps rank compounds according to their affinities for each isozyme.
• Affinities for Carbonic Anhydrase Isozymes by Various Techniques tool, which enables visual comparison of affinities obtained by alternative orthogonal biophysical methods.
• Enthalpy-Entropy Compensation Plot and Table, which provides enthalpy-entropy distribution for each compound.

PLBD-Tools extends the strict text-based tabular representation of binding thermodynamics data with enhanced table views and interactive plots. The PLBD-Tools suite consists of compound-oriented applications, all of which support filtering by compound name fragments.

In the plotting tools, including Affinities for Carbonic Anhydrase Isozymes by Various Techniques and Enthalpy-Entropy Compensation Plot and Table, users can select which protein groups to display. Numerical values in table cells or corresponding data points in plots can be represented through color coding. Detailed information about selected data points is displayed in a panel on the left-hand side.

For quick help with the tool web application element, one can use hints in the tooltips. A more comprehensive explanation of the controls and value-containing elements is organized as a separate pop-up window.

PLBD-Tools enables fast and convenient navigation within the binding database. Visualization of binding parameters, using color-coded tables or graphical representations, enables rapid identification of the most strongly interacting compounds. The results are presented in Table 1. In this table, we automatically extracted the compounds that bind strongest to each CA isozyme. When the database has a stronger compound, it will be updated in such searches. Both the observed and intrinsic K_d_ values are provided. The observed values are determined directly by any experimental technique, while the intrinsic values are corrected according to the binding-linked protonation-deprotonation reactions.^16^

**Table 1:** Observed and intrinsic dissociation constants of selected compounds binding to CA isozymes at 37°C, as determined by the fluorescent thermal shift assay

Isozyme	Compound	*K*_*d*,*obs*_ (nM)	*K*_*d*,*int*_ (nM)
CA I	VD11-61	0.025	0.00066
CA II	VD10-49	0.79	0.045
CA III	TFMSA; TFS	1,000	220
CA IV	EA3-2	1.4	0.0042
CA VA	E2	0.13	0.0016
CA VB	VD12-05	0.050	0.0017
CA VI	TFMSA; TFS	14.	1.2
CA VII	VD10-49	0.22	0.011
CA IX	MKV-466	0.0038	0.000053
CA XII	MKV-466	0.050	0.00094
CA XIII	MKV-466	0.25	0.011
CA XIV	VD10-49	0.50	0.025

These compounds exhibit the strongest intrinsic binding to the isozymes.

### Compound Browser tool

The Compound Browser tool uses the “compound” database view as its data source. The tool enables browsing all table rows within a single continuous interface and provides additional functionality beyond the standard RestfulDB middleware capabilities.

The user can view the compound structural formula in three different ways (Figure 2).

• A dynamically rendered structural formula generated from the SMILES string using SmilesDrawer, a JavaScript-based external package.^20^
• A structural formula generated from the SMILES string using the RDKit package^26^ on the server side.
• The original image stored in the database as a BLOB.

**Figure 2:**
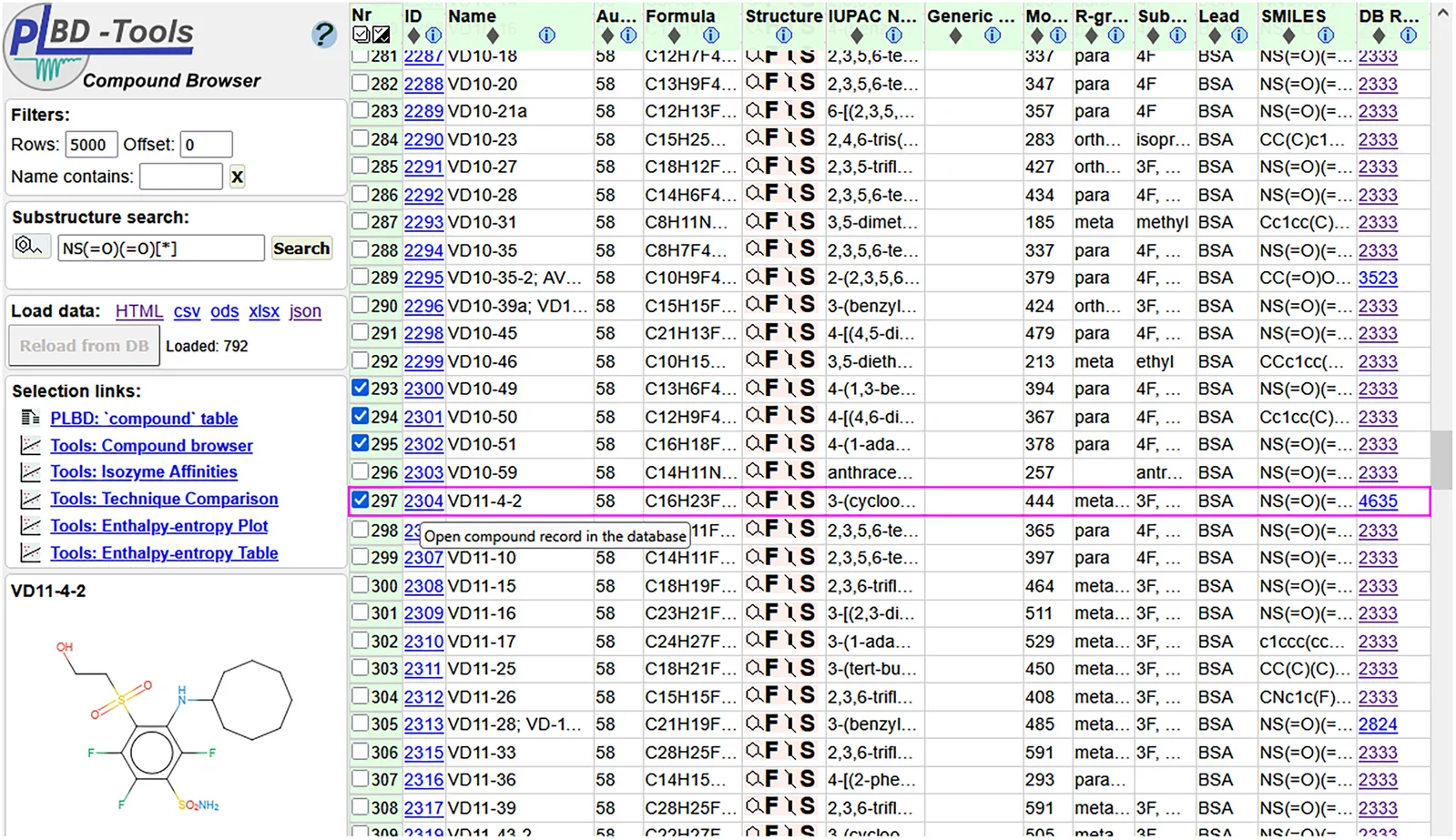
Compound Browser tool enables convenient review of available compound chemical structures, SMILES, and links to each compound batch in the database.

For alternative formula representations, the user can click the icons in the “Structure” column. Clicking the “F” icon updates the left-side card to display the original formula drawing stored in the PLBD field “compound.formula_file.” Clicking the “S” icon updates the left-side card to display a formula drawing generated by the PLBD-Tools RDKit package.

#### Substructure search

The substructure search function employs the RDKit package.^26^ The search is performed by submitting a SMARTS pattern representing the query fragment to the RDKit package acting on the PLBD-Tools server. The SMARTS pattern can be entered manually in the input field or generated using the “Chem Composer” widget from the Kekule.js library.

In substructure search results, the matched fragment is highlighted within the rendered structure. Using Ctrl + mouse click on the icons in the “Structure” column opens structural formula drawings in a separate panel at the bottom of the browser window: the original-size image from the “formula_file” field or an enlarged version generated by RDKit (Figure 3). This feature can be used to verify compound structures and compare stored images with dynamically generated representations.

**Figure 3:**
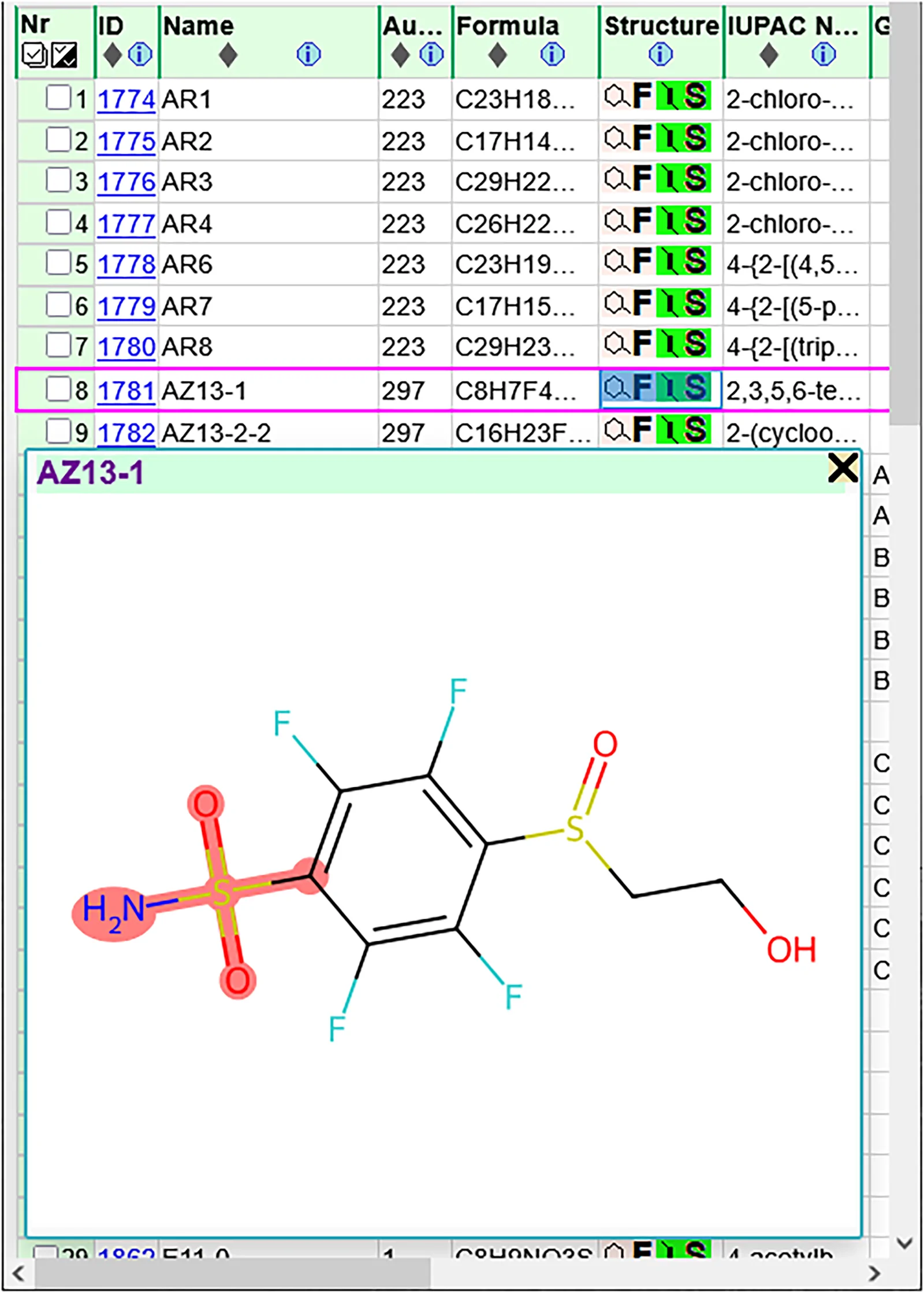
Searching for a chemical structure fragment in the Compound Browser tool.

### Compound Affinities for CA Isozymes tool

The tool presents a numerical table of thermodynamic parameters for sulfonamide compound binding to CA isozymes, extracted from the PLBD, using a user-defined color scale. Depending on the selected parameter, it uses one of the following database views as the data source: kd_obs_tsa_all_cas, kd_int_tsa_all_cas, or kb_int_tsa_all_cas (observed or intrinsic K_d_ or *K*_*b*_, for all CA isozymes). The user can choose to display either observed or intrinsic thermodynamic parameters of compound binding. The data may be presented as binding or dissociation constants or, alternatively, as standard Gibbs free energies of binding.

The color scheme follows conventions commonly used in previously developed specialized software: red indicates stronger binding, whereas blue indicates weaker binding, relative to a defined midpoint. The user can configure the coloring ranges for the selected thermodynamic parameter, including the midpoint, as well as the minimum and maximum values, and the number of color steps. The number of steps is applied symmetrically to both the negative and positive ranges.

Rows of the exposed database table can be sorted in ascending and descending order by the value of each column. Sorting can be multi-stage, and the order of the sorting stage is shown by a number following the control element. The compact table view, combined with multi-column sorting and customizable color scaling based on a selected thermodynamic parameter, enables efficient and intuitive analysis of the binding properties across a set of compounds.

The tool is useful to discover how a compound of variable molecular size could fit into the isozyme containing the active site pocket able to accommodate a compound of a particular but not greater size. A series of di-meta-substituted benzenesulfonamides bound with increasing affinity upon increased compound size until the available space limit is reached, resulting in an activity cliff (Figure 4).^8^ Such trends could be visualized using the tool.

**Figure 4:**
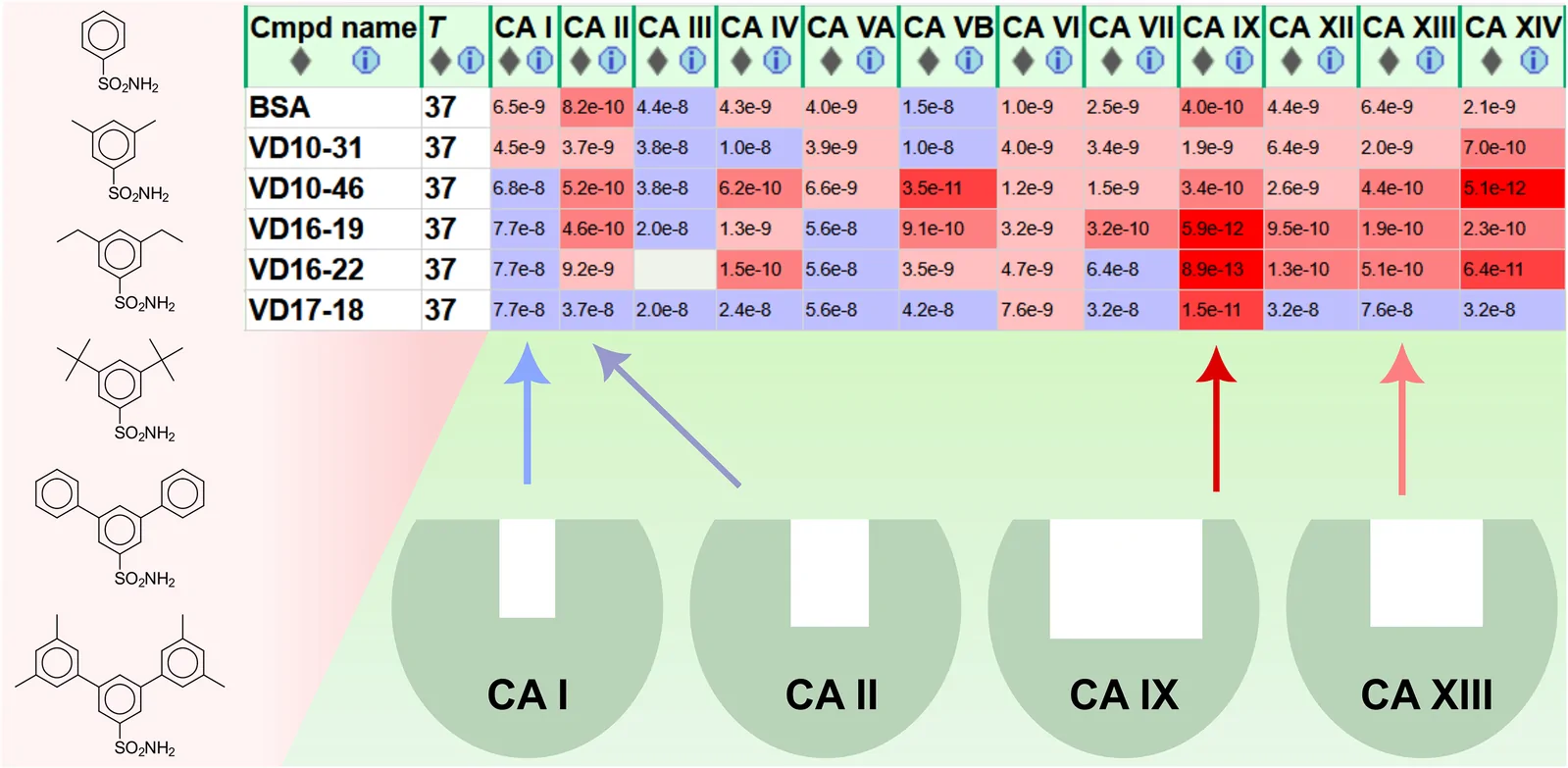
Comparing meta-substituted compound intrinsic dissociation constants in the Isozyme Affinities tool. Isozymes have variable-size binding pockets and are able to fit compounds of corresponding size, as visualized in the dissociation constant values for closely related compounds of increasing molecular size. Affinity abruptly falls when the compound can no longer fit in the pocket, an example of the affinity cliff.

### Affinities for Carbonic Anhydrase Isozymes by Various Techniques tool

In this tool, we compare the results obtained by various biophysical techniques used to measure compound-protein affinities. We currently include four techniques: TSA, ITC, SFA, and SPR.

The Affinities for Carbonic Anhydrase Isozymes by Various Techniques tool uses “all_method_kd_obs_stats_log10” or “all_method_kd_int_stats_log10” database views as data sources, depending on the observed or intrinsic dissociation constant to represent.

The Affinities for Carbonic Anhydrase Isozymes by Various Techniques tool (Figure 5) depicts the data of thermodynamic parameters obtained by different instrumental techniques in a 2D plot. The user can choose between four thermodynamic parameters to show: K_d,*obs*_, Δ*G*_*obs*_ , K_d,*int*_, and Δ*G*_*int*_. One of the four commonly employed instrumental techniques is freely selectable on both the *x* and *y* axes.

**Figure 5:**
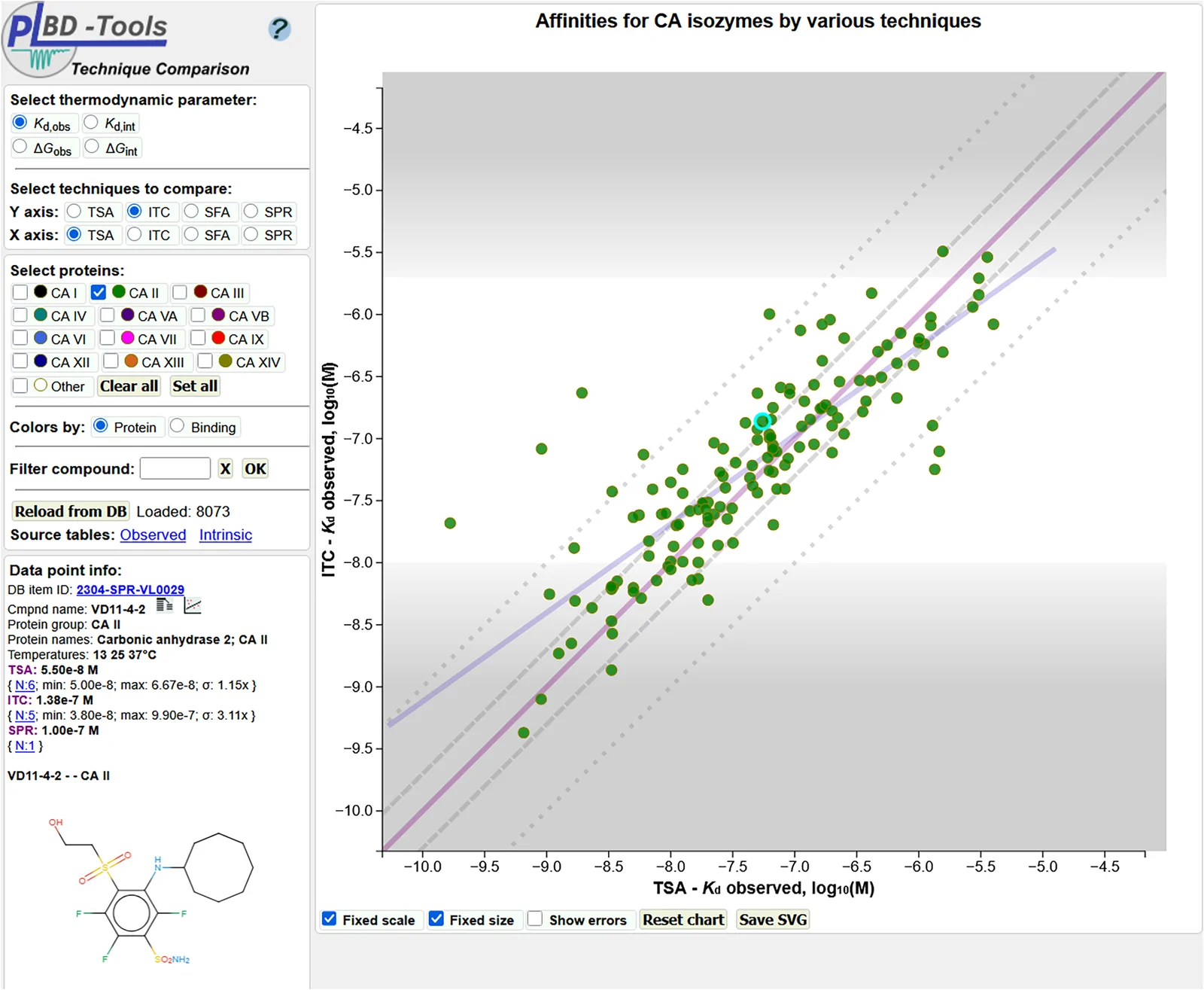
Comparison of TSA and ITC experimental data in the Technique Comparison tool. Comparison is available for four techniques, including SFA and SPR.

Additional graphical window control, the “Fixed scale” check box, enables switching between preset axis ranges and a dynamic range adopted to visible data points.

#### Reliable experimental measurement window

The white region in the plot (Figure 5) represents the reliability window, i.e., the range of dissociation constants that can be determined accurately with a given method. This window defines the region of concentrations and affinities where the experimental response remains sufficiently sensitive to changes in binding. In practical terms, it indicates the conditions under which a method can reliably distinguish between different interaction affinities. Outside this window, the dissociation constants cannot be determined accurately. Thus, the white background in the plot visually highlights the parameter space where a given method performs optimally and yields the most reliable results.^27^

The dynamic linear regression analysis of viewable data points is performed, and the results are exposed in the left-side panel, “Current graph data points info,” alongside the value ranges.

### Enthalpy-Entropy Plot and Table

The Enthalpy-Entropy Compensation Plot and Table tool uses a single “enthalpy_int_stats” database view as the data source.

In the enthalpy-entropy compensation plot for inhibitors (Figure 6), different colors represent binding data to various CA isozymes. Most compounds bind to CAs due to favorable enthalpy and favorable entropy contributions, where one or the other is dominant. However, a few were bound by enthalpy-opposed or entropy-opposed thermodynamics. The user can choose between TSA and ITC experiments to calculate intrinsic Δ*G*, the temperature of the experiments, protein groups to see, and filter compounds by name fragment.

**Figure 6:**
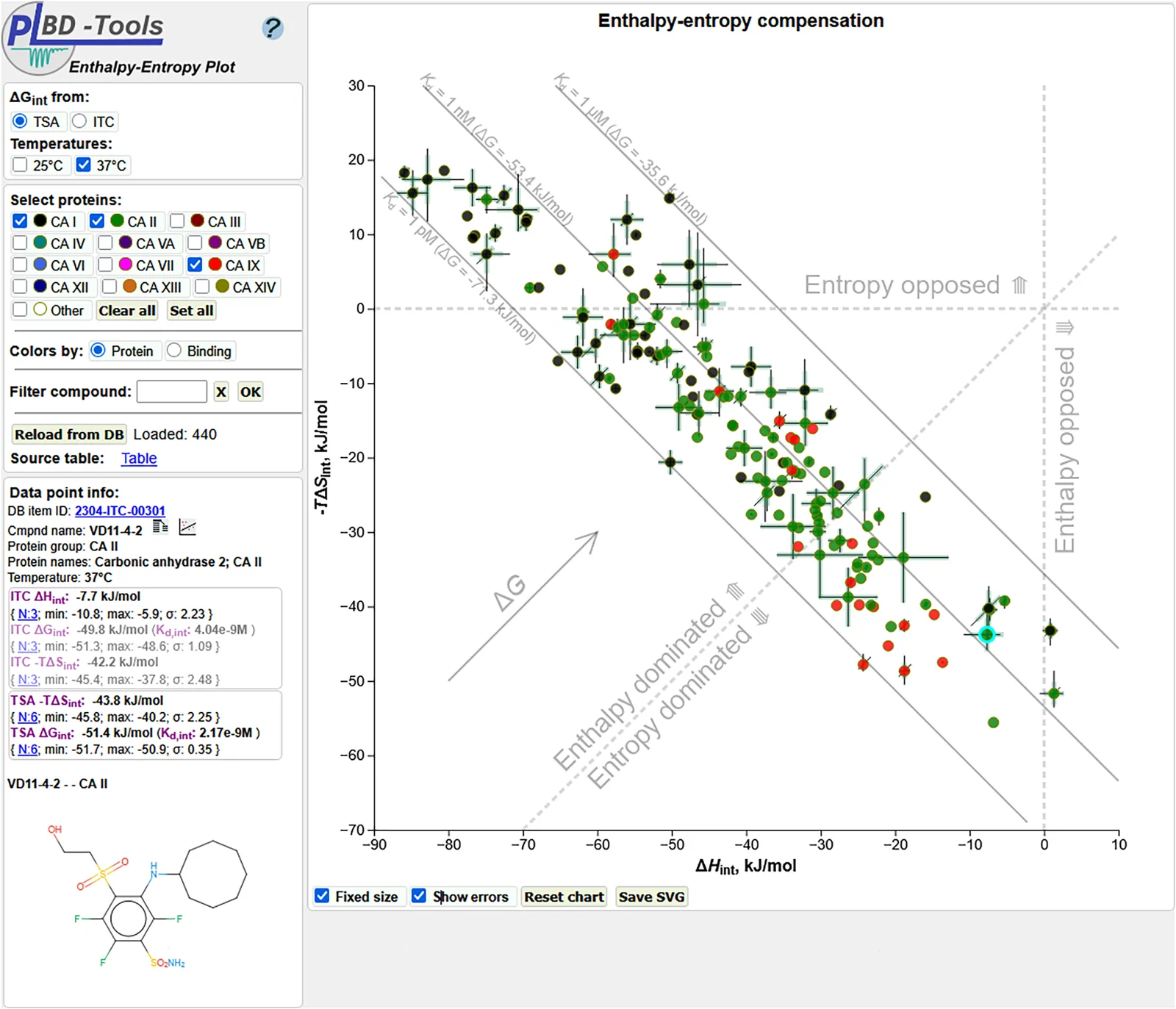
Enthalpy-Entropy Compensation Plot shows the intrinsic enthalpies and entropies, and the diagonal lines show the Gibbs energies. Error bars show standard deviations when two or more repeats are available for each measurement in the database.

The Δ*H*_*int*_ and −*T*Δ*S*_*int*_ scale ranges are fixed in the “Enthalpy-Entropy” plot, covering an entire range of possible values. The diagonal lines represent different *ΔG* values, ranging from 1 μM to 1 pM, thus spanning a millionfold difference in affinity.

In the enthalpy-entropy compensation table (Figure 7), the same data are represented in table form. Every point in the plot corresponds to a four-cell block in the table where the background is colored by thermodynamic parameter value.

**Figure 7:**
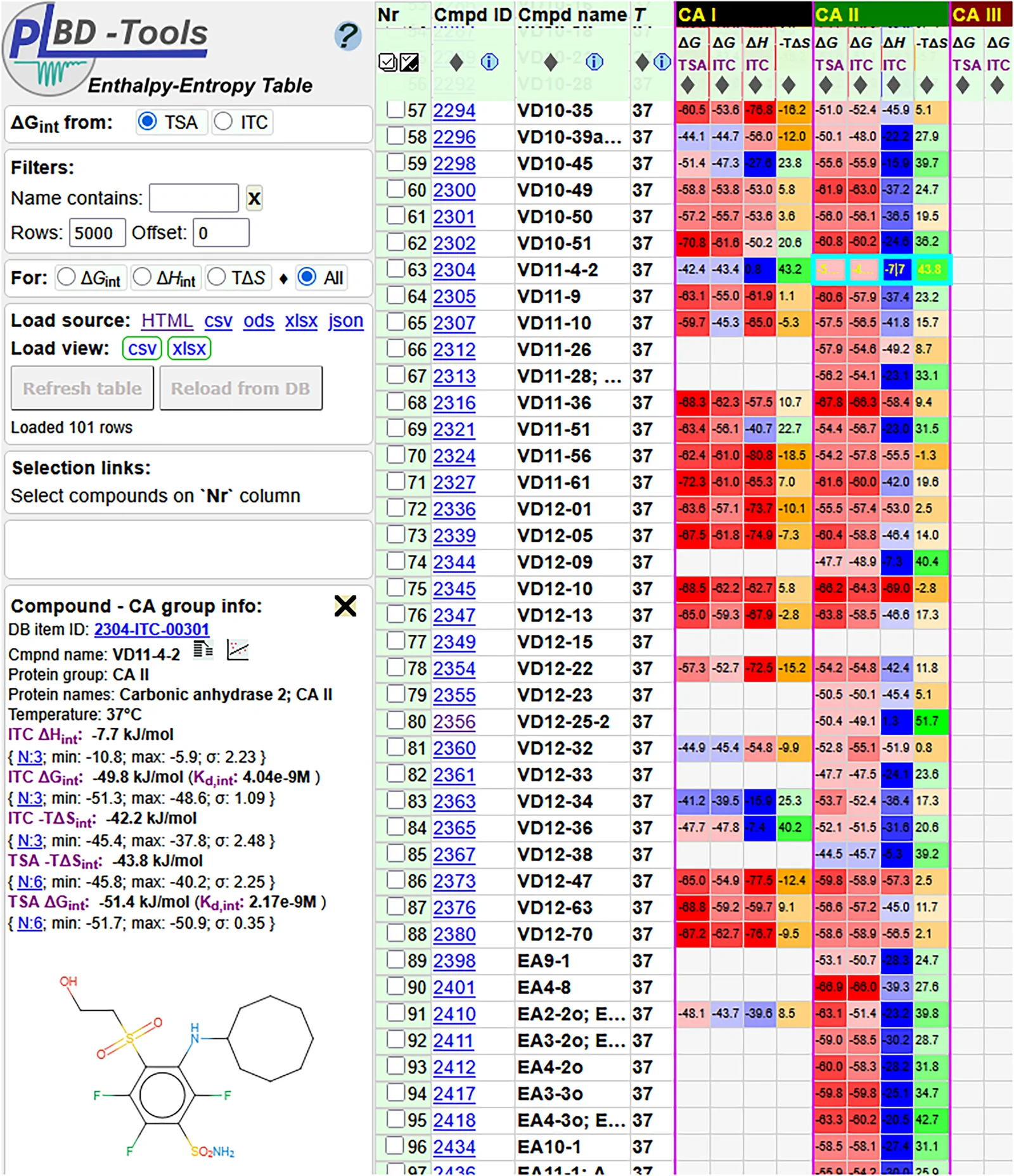
The enthalpy-entropy table lists the compounds and their intrinsic Gibbs energies of binding, as obtained by two techniques, TSA and ITC, intrinsic enthalpies obtained by ITC, and the entropy-temperature product obtained by subtracting the technique-selected Gibbs energy.

### Common user interface and features available for all PLBD-Tools

Currently, two types of tools are created: tables and plots.

The table tools load and display the entire range of data records without pagination. They present a slightly reduced set of columns, and the table cells are compact, allowing more information to be displayed within the limited space of a browser window.

The tools provide quick access to commonly used RestfulDB middleware functions, such as sorting by column values and exporting visible data in various formats. More advanced features, including multi-stage filtering for each field (column), are available only on the RestfulDB pages.

The primary purpose of the table tools is to facilitate browsing and selecting chemical compounds for *xy* scatter plots. Compound selection is based on binding thermodynamics and chemical structure.

#### Selecting multiple rows (compounds)

Every row in the table represents properties of an individual chemical compound. For further analysis, the user can pick the items (compounds) of interest by selecting corresponding table rows. As a result, the collection of web links appears in the “Selection links” left-side panel, pointing to pages with information related to only the restricted set. The first link leads to the “compound” table with full information on selected compounds using the relational database and RestfulDB middleware capabilities. Other links provide a complete set of PLBD-Tools concentrated on selected chemical compounds.

#### Filters card

The “Filters” card contains only three essential items from RestfulDB capabilities. In the table tools, the whole database table is loaded from the server by default. When necessary, a limited number of data rows can be loaded from the database by setting URL parameters, rows, and offset in the corresponding elements. Filtering compounds by name fragment can be done by filling in the “Name contains:” element.

#### Load data card

The “Load data” card provides the number of currently loaded data records (rows) and links for quick download of the tables in various file formats. Data points represented in the graph window can be downloaded in CSV and XLSX formats.

#### Reduced set of database table fields (columns)

For better database table content visibility, nonessential data columns (database table fields) are hidden. The list of hidden table columns is kept in the JavaScript constant “initOffColumns.”

#### Column controls

Column headers contain “sort buttons” and “info links.” Search buttons cyclically change between three modes: “no sort,” “ascending,” and “descending.” “Info links” leads to a database XML description (XSL transformed), expanding and focusing on the corresponding table and field description.

#### Multi-stage sort of columns

Rows of the exposed database table can be sorted in ascending and descending order by the value of each column. Sorting can be multi-stage, and the order of the sorting stage is shown by a number following the control element. The control element shape changes cyclically, in the sequence ascending-descending-no sort.

#### Dynamic row highlight and chemical structure draw

PLBD tools are compound-oriented applications. Following this concept, multiple rows representing one compound are highlighted and selected simultaneously. In the “Isozyme Affinities” tool, rows with different experimental temperatures for the same compound are highlighted simultaneously. In addition to table row(s) highlighting, the name and structural formula of the corresponding compound are exposed in the left-side panel. The compound structural formula is drawn dynamically from SMILES using SmilesDrawer external software.^20^

#### Reload from DB button

In contrast with the RestfulDB interface, where all changes occur in series and require waiting for data exchange with the server, table tools requests all changes simultaneously. “Reload from DB” button activates after the first change of the “Filters:” element or any of the column sort buttons.

#### Left-side panels

In the first left-side panel, the user can choose a thermodynamic parameter and instrumental technique to compare (“Comparison of Techniques” tool) or the intrinsic Gibbs energy calculation source and temperatures of experiments (“Enthalpy-Entropy” tool). The next setting panel provides controls for filtering displayed experimental data points by CA groups and compounds. The user can select a desirable set from 12 available CA groups and the way the data points are colorized. Additionally, compound filtering by the name fragment is available. Using the “Reload from DB” button, users have the possibility to forcibly request source table data loading to the web application. The “Table” link causes the source table to open in a new browser tab using RestfulDB HTML representation. The next two left-side panels are responsible for providing on-the-fly information about visible data points in the graphical window. The “Data point info” card shows information on the selected data point: compound name, protein name, protein group, thermodynamic parameter values, and compound structural formula. Some items in this left-sided card are supplemented with direct links to the corresponding database records. The “Current graph data points info” card provides count and value ranges of the currently viewable data point set.

#### Graphical window

For plot tools, the D3 JavaScript data visualization library^22^ is used. Data points are shown as circles colored in two ways: by protein group or binding strength, as in the Isozyme Affinities tool. The graphical window features zooming and dragging with standard mouse controls. Moving the mouse cursor among data points causes the appearance of tooltips with short information on a particular item. Clicking the left mouse button selects the data point, and explicit information appears in the left-side card.

#### Graphical window controls

Controls at the bottom of the graphical window complement the controls of the left-side panels. The “Fixed scale” checkbox switches between a fixed (maximal, default) axis scale range and a dynamic one when changing browser window size. The “Show errors” checkbox switches on error bars on data points where the corresponding protein-ligand pair represents more than one experiment done. The “Reset chart” button returns the zoomed graph window to its start appearance. Pressing the “Save SVG” button leads to the generation of an SVG file from the nonzoomed current graph window. The file is downloaded or viewed depending on your browser settings.

#### Graph data area features

The user can zoom and pan the graph area using standard OS techniques with a mouse or a touchscreen. These functionalities are realized using the D3.js library as SVG transformations. By positioning the mouse cursor on the data point, the user can obtain a tooltip with brief information. It includes the names of the protein group and ligand used, temperature, and *x* and *y* values. More comprehensive information appears in the left-side panel when a particular data point is selected by mouse click.

#### Error bars

PLBD-Tools plots, including Affinities for Carbonic Anhydrase Isozymes by Various Techniques and Enthalpy-Entropy Compensation Plot, support the display of error bars for individual data points. Error bars are shown when more than one experimental measurement is available.

Thin black lines indicate the full range of measured values (from minimum to maximum) along each axis, while green bars represent the standard deviation of the measurements.

#### Edge ligand concentrations

Some chemical compounds possess limited aqueous solubility. This makes it impossible to determine low-binding affinities that require high compound concentrations.^5^ In the Affinities for Carbonic Anhydrase Isozymes by Various Techniques tool, such cases are depicted as half-filled circles (Figure 8).

**Figure 8:**
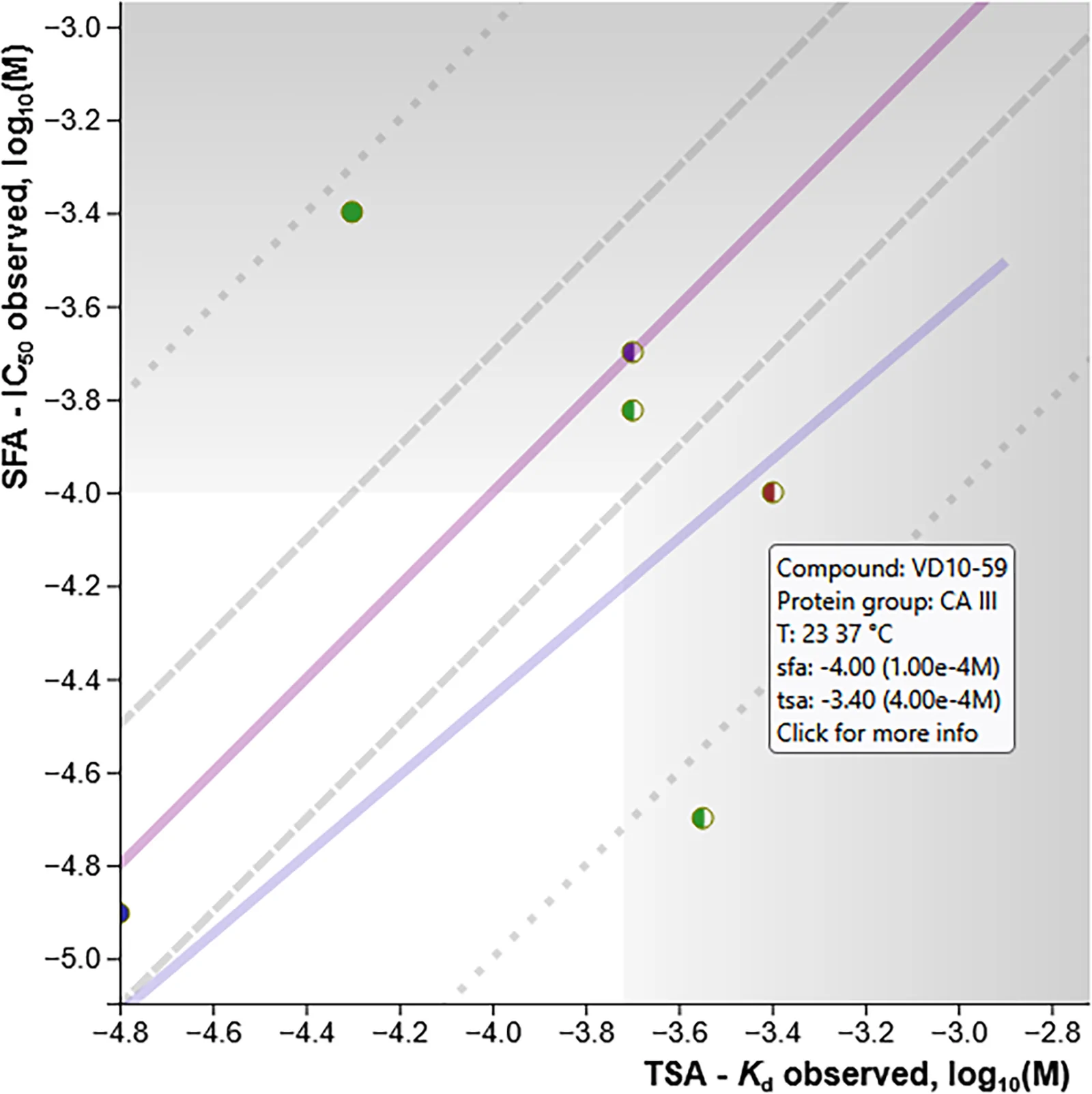
Ligand maximum used concentration data points showing that the true values could be beyond the range of the white area, where the measurements fall within the accurate determination range. Half-filled circles represent such situations, where the real dissociation constant could be beyond the shown value.

## Discussion

In this manuscript, we presented a set of web-based tools to analyze and visualize the data provided in the PLBD. The manuscript is primarily methodologically oriented, intended to help a potential user of the database visualize and analyze the vast amount of data. However, while using the tools, we have discovered or noticed some new trends and tendencies in the data. Most notably, the enthalpies and entropies of compound binding spread throughout a wide range, and structurally similar compounds sometimes bind with highly opposing enthalpy- or entropy-dominated signatures. We could not see a generalizable trend. However, some tendencies will be discussed in future studies. Second, it was convenient to arrange compounds according to molecular size and see activity cliffs when the isozyme pocket could not accommodate a compound of increased size, leading to an abrupt drop in affinity due to steric hindrance.

We would like to put the PLBD and its analysis tools in the context of existing databases. The Database Commons catalog^28^ lists more than 7,300 biological databases. Among this vast collection of resources, only a limited number provide quantitative protein-ligand interaction affinity data. Particular attention should be given to databases that not only support online searching and browsing but also allow users to download complete data sets for local installation, custom analyses, and integration into their own research workflows and computational projects. Below, we provide a brief description of available databases that provide similar protein-ligand binding data to the PLBD.

• BindingDB^29^^,^^30^ was the first binding affinity database to provide experimental affinity measurements for every included entry. Data are manually and automatically curated from the scientific literature, patents, other databases, and direct submissions by experimentalists. The database is updated monthly and can be downloaded as a complete dump in MySQL or Oracle DB formats, together with several predefined data subsets. BindingDB is implemented as a relational database consisting of 32 tables.
• PDBbind^31^ provides experimental binding affinity data for biomolecular complexes deposited in the Protein Data Bank (PDB).^32^ Users can download separate data sets for protein-ligand, protein-protein, protein-nucleic acid, and nucleic acid-ligand complexes. The protein-ligand data set currently contains approximately 19,000 records with affinity measurements.
• ChEMBL^33^ is a manually curated database of bioactive molecules with drug-like properties. Periodic releases are available as complete dumps in SQLite, MySQL, PostgreSQL, and Oracle DB formats. The ChEMBL database comprises 74 relational tables. Binding parameters originate from multiple sources and are represented using different notations and units. For example, the dissociation constant, K_d_, may be denoted as *Kd*, *Kdiss*, or *KD*50 and reported in more than 50 distinct concentration units.
• BioLiP2^34^ is designed to extract biologically relevant protein-ligand interactions from structures deposited in the PDB.
• Psychoactive Drug Screening Program *K*_*i*_ Database^35^ is a collection of inhibition constants for psychoactive compounds. It contains affinity values (*K*_*i*_) for a large number of drugs and drug candidates interacting with G protein-coupled receptors, ion channels, transporters, and enzymes. The database consists of a single table containing nearly 100,000 *K*_*i*_ measurements together with protein, ligand, and literature reference information.
• Binding MOAD^36^ (Mother of All Databases; currently not accessible) was a curated, structure-based resource of protein-ligand complexes derived from the PDB, enriched with experimentally determined binding affinity data collected from the primary literature. Established in the early 2000s, it has served as a benchmark data set for studies in structure-based drug design, molecular docking, scoring function development, and binding affinity prediction. In its final public release, Binding MOAD contained over 40,000 protein-ligand complexes and thousands of unique ligands with associated affinity measurements. Although the project has been formally sunset and is no longer actively updated, the archived data set remains a valuable reference resource for computational and structural biology research.
• Drug Target Commons,^37^ which is currently not available.

Statistics of protein-ligand binding parameters available in currently maintained affinity databases are summarized in Table 2.

**Table 2:** Public protein-ligand binding databases giving quantitative affinity data

Database	Year, version	Parameters	No. of values	Availability of the raw data
BindingDB^29^^,^^30^	2026	overall	3.2 M	indirect, through publications
		*K*_d_	127,840	
		*K*_*i*_	621,720	
		IC_50_	2,168,897	
		EC_50_	266,801	
		Δ*H*	1,701	
PDBbind^31^	2020 R1	overall	19,443	indirect, through publications
		*K*_d_	7,249	
		*K*_*i*_	5,004	
		IC_50_	7,190	
ChEMBL^33^	2026 v.37	overall	5 M	indirect, through publications
		*K*_d_, *K*_*b*_	214,972	
		*K*_*i*_	763,848	
		IC_50_	3,608,930	
		EC_50_	635,756	
BioLiP2^34^	2025	overall	19,181	no data
		*K*_d_	5,598	
		*K*_*b*_, *K*_*a*_	156	
		*K*_*i*_	6,530	
		IC_50_	9,645	
		EC_50_	1,272	
PDSP^35^	2025	*K*_*i*_	93,103	indirect, through publications
PLBD^38^	2026	overall	10,899	yes, files and images
		*K*_d_	9,686	yes
		IC_50_	91	yes
		Δ*H*	1,122	yes

Free-access data were collected in 2026.

Publicly accessible databases containing quantitative protein-ligand binding data have become indispensable resources in modern drug discovery and chemical biology. Major repositories comprise extensive collections of experimental measurements spanning numerous protein families and thousands of chemically diverse compounds. Although these databases provide valuable access to affinity, thermodynamic, and kinetic information, search results are commonly presented in plain-text or conventional tabular formats. While such representations are effective for data storage and retrieval, they may limit the rapid identification of trends, outliers, selectivity patterns, and structure-activity relationships, particularly when large data sets are examined.

To facilitate PLBD data exploration, a collection of specialized database views has been developed. These views provide convenient access to the underlying PLBD data either through standard RestfulDB interfaces or through the PLBD-Tools applications.

The PLBD-Tools web application suite adopts a different approach by emphasizing visual data exploration and interpretation. At present, the platform focuses on a single protein family, human CAs, encompassing all 12 catalytically active isozymes and 792 compounds with experimentally determined binding data. Although narrower in scope than general-purpose affinity databases, this specialization enables the implementation of visualization strategies tailored to a well-defined biological system.

A distinctive feature of PLBD-Tools is the presentation of experimental data through interactive plots and color-encoded tables. In these representations, each table cell conveys not only numerical information but also additional visual information through color gradients corresponding to the magnitude of the measured parameter. Such dual encoding enables rapid identification of affinity trends, isozyme selectivity, thermodynamic signatures, and kinetic characteristics across multiple ligands and protein isoforms. Visual representations substantially reduce the effort required to compare large numbers of measurements and facilitate recognition of patterns that may remain less apparent in conventional text-based presentations.

These observations demonstrate that even comparatively focused data sets can benefit considerably from advanced visualization approaches. By integrating quantitative information with intuitive graphical representations, PLBD-Tools enhances data accessibility and supports hypothesis generation, compound prioritization, and the analysis of ligand-protein interaction patterns. Future developments may extend the platform to additional protein families and incorporate novel visualization and analytical capabilities, further increasing its utility for exploring protein-ligand binding data. One of the major goals in structure-thermodynamics correlation research is not only correlations between compound chemical structures and binding thermodynamics but also correlations between protein 3D structures and binding thermodynamics. We plan to introduce a tool in the future that would help visualize and correlate structural differences in both compounds and proteins and the thermodynamic parameters of binding.

## Data and code availability

The data are available online at plbd.org. The PLBD-Tools web application code is available at GitHub: https://github.com/Biothermodynamics-and-Drug-Design/plbd-tools.

## Acknowledgments

The authors acknowledge Lina Baranauskienė and Asta Zubrienė for requests of table views and features and Marius Gedgaudas for GitHub assistance.

## Author contributions

D.L. conceived the idea of the PLBD-Tools, programmed the web applications, set up the server, analyzed PLBD data, and wrote and edited the manuscript. D.M. proposed the table and chart design and tested the web applications. Both authors have read and approved the final version of the manuscript.

## Declaration of interests

D.M. authored several patents and pending patent applications for CA inhibitors.

## Declaration of generative AI and AI-assisted technologies in the manuscript preparation process

During the preparation of this work, the authors used ChatGPT for language editing. The authors reviewed and edited the output as needed and take full responsibility for the content of the published article.
